# Changes in Atlantic major hurricane frequency since the late-19th century

**DOI:** 10.1038/s41467-021-24268-5

**Published:** 2021-07-13

**Authors:** Gabriel A. Vecchi, Christopher Landsea, Wei Zhang, Gabriele Villarini, Thomas Knutson

**Affiliations:** 1 Geosciences Department, Princeton University, Princeton, NJ USA; 2 High Meadows Environmental Institute, Princeton University, Princeton, NJ USA; 3 National Hurricane Center, National Weather Service, National Oceanic and Atmospheric Administration, Miami, FL USA; 4 Department of Plants, Soils and Climate, Utah State University, Logan, UT USA; 5 IIHR-Hydroscience & Engineering, University of Iowa, Iowa City, IA USA; 6 Geophysical Fluid Dynamics Laboratory, National Oceanic and Atmospheric Administration, Princeton, NJ USA

**Keywords:** Atmospheric dynamics, Climate change, Attribution

## Abstract

Atlantic hurricanes are a major hazard to life and property, and a topic of intense scientific interest. Historical changes in observing practices limit the utility of century-scale records of Atlantic major hurricane frequency. To evaluate past changes in frequency, we have here developed a homogenization method for Atlantic hurricane and major hurricane frequency over 1851–2019. We find that *recorded* century-scale increases in Atlantic hurricane and major hurricane frequency, and associated decrease in USA hurricanes strike fraction, are consistent with changes in observing practices and not likely a true climate trend. After homogenization, increases in basin-wide hurricane and major hurricane activity since the 1970s are not part of a century-scale increase, but a recovery from a deep minimum in the 1960s–1980s. We suggest internal (e.g., Atlantic multidecadal) climate variability and aerosol-induced mid-to-late-20th century major hurricane frequency reductions have probably masked century-scale greenhouse-gas warming contributions to North Atlantic major hurricane frequency.

## Abstract (web summary)

How tropical cyclones have varied in intensity and frequency in the past is not well known as longer records are rare. Here, the authors show that changes in observing practices explain the recorded century scale increase in Atlantic major hurricane frequency, and recent increases are not part of a century-scale trend.

## Introduction

Tropical cyclones (TCs) are of intense scientific interest and are a major threat to human life and property across the globe^1–3^. Of particular interest are multi-decadal changes in TC frequency arising from some combination of intrinsic variability in the weather and climate system, and the response to natural and anthropogenic climate forcing^4–6,14–25^. Even though the North Atlantic (NA) basin is a minor contributor to global TC frequency, Atlantic hurricanes (HUs) have been the topic of considerable research both because of the long-term records of their track and frequency that exist for this basin, and because of their impacts at landfall. It is convenient and common to consider Saffir-Simpson Categories 3–5 (peak sustained winds exceeding 50 ms^−1^) HUs separately from the overall frequency, and label them major hurricanes, or MHs. Historically, MHs have accounted for ~80% of hurricane-related damage in the United States of America (USA) despite only representing 34% of USA TC occurrences^1^.

Globally, models and theoretical arguments indicate that in a warming world the HU peak intensity and intensification rate should increase, so that there is a tendency for the fraction of HU reaching high Saffir-Simpson Categories (3, 4, or 5) to increase in models in response to CO_2_ increases, yet model projections are more mixed regarding changes in the frequency of MHs in individual basins (e.g., NA)^6,20–22,25–30^. Homogenized satellite-based TC intensity observations since the early 1980s show an increase in the fraction of MH to overall TCs both in the NA and globally^14^, and there has also been a documented increase since the 1980s in the fraction of global and NA HU that undergo rapid intensification^15^. Theoretical arguments, modeling studies, and observational analyses indicate that the overall frequency of TCs and their intensity across the tropics, and for Atlantic HUs in particular, may vary differently and exhibit distinct connections to climate drivers^14,15,25–32^. There is substantial spread in model projections of the 21st century response of both overall NA HU frequency and of the response of the frequency of the most intense NA HUs^6,20–22,25–30^. However, the connection between recent recorded multi-decadal changes in NA HU activity and 21st century HU projections is complicated by the fact that recent changes (e.g., since the 1970s) in NA HU and MH activity likely contain a substantial contribution from internal climate variation or non-greenhouse gas forcing^16–23^.

Has there been a century-scale change in the number of the most intense hurricanes in the North Atlantic? Analyses of longer records (i.e., going back into the 19th century) of NA HU and MH frequency provide an additional lens with which to interpret both recent HU activity changes and projections of future hurricane activity. The North Atlantic Hurricane Database version 2 (HURDAT2; ref. ^33^) provides records of NA HU activity going back to 1851—a nearly 170-year record of HU activity. Using HURDAT2, one can explore secular changes in aggregate statistics of NA HU activity, such as the annual number of HU and MH strikes in the USA and the annual number of HUs and MHs in the Atlantic (or basin-wide HU and MH frequency). The USA HU strike record we use includes storms for which either hurricane strength, or *v*_max_ ≥ 33 ms^−1^, or major hurricane strength, or *v*_max_ ≥ 50 ms^−1^, winds impacted the continental USA from the Atlantic or Gulf of Mexico, so this record includes storms for which the center did not cross onto land.

Due to changes in observing practices, severe inhomogeneities exist in this database, complicating the assessment of long-term changes^7–13^. In particular, there has been a substantial increase in monitoring capacity over the past 170 years, so that the probability that a HU is observed is substantially higher in the present than early in the record^10^; the recorded increase in both Atlantic TC and HU frequency in HURDAT2 since the late-19th century is consistent with the impact of known changes in observing practices^7–12^. Major hurricane frequency estimates can also be impacted by changing observing systems^13^.

We here show that recorded increases in NA HU and MH frequency, and in the ratio of MH to HU, can be understood as resulting from past changes in sampling of the NA. We build on the methodology and extend the results of ref. ^10^ to develop a homogenized record of basin-wide NA HU and MH frequency from 1851–2019 (see Methods Section), this homogenized record indicates that the increase in NA HU and MH frequency since the 1970s is not a continuation of century-scale change, but a rebound from a deep minimum in the late 20th century.

## Results

### Recorded century-scale NA hurricane changes

**Fig. 1:**
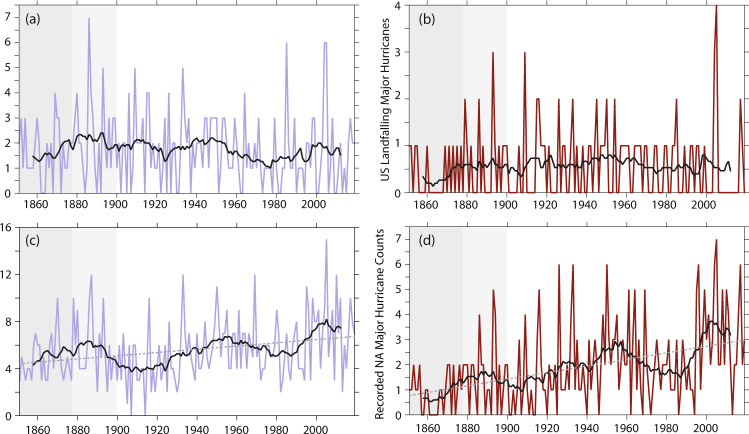
Recorded USA hurricane strikes and basin-wide frequency. Time-series of annual (colored lines) and 15-year running average (black lines) count of hurricanes (panels **a** and **c**; maximum wind speed ≥33 ms^−1^) and major hurricanes (panels **b** and **d**; maximum wind speed ≥50 ms^−1^) either striking the mainland United States of America (USA; panels **a** and **b**) or in the whole North Atlantic basin (panels **c** and **d**) from version 2 of the North Atlantic Hurricane Database (HURDAT2, ref. ^33^). Dark gray background shading between 1851–1877 indicates the period before the United States Signal Corps was tasked with recording all tropical cyclones in the North Atlantic (ref. ^35^), light gray background shading between 1878 and 1900 indicates the time before which it is estimated that all hurricanes striking the USA would have been recorded (ref. ^36^).

Neither the number of HU nor MH striking the USA are dominated by century-scale changes between 1851 and 2019, although each exhibits substantial year-to-year and decadal fluctuations (Fig. 1a, b). There is a decrease in the recorded number of USA HU strikes, that may be statistically significant for certain periods (e.g., Table 1) or depending on the statistical model used^34^. Hurricane data are available from 1851 onwards, but even for USA-striking HUs and MHs there are likely to be inhomogeneities including undersampling over this period. We show the data for the full 1851–2019 record, but highlight the pre-1878 era with dark gray background shading—as 1878 was the year in which the U.S. Signal Corps began systematic efforts to catalog all Atlantic HUs^35^. Furthermore, it is likely that U.S. coastal regions did not become sufficiently well-populated to fully monitor US-striking HUs and MHs until at least the year 1900 (ref. ^36^), so we highlight the 1878–1900 period with lighter gray shading in our figures. Basin-wide NA HU and MH frequency shows substantial year-to-year and multi-decadal variation, some of which is reflected in U.S. striking frequency (Fig. 1).

**Table 1:** Measures of secular change in hurricane frequency.

	Time-dependence of the rate parameter of Poisson regression (λ) (1/century)			
	1851–2019	1878–2019	1900–2019	1980–2019
USA hurricane strikes	−0.139 (*p* = 0.25)	−0.307 (*p* < 0.05)**	−0.198 (*p* = 0.33)	0.570 (*p* = 0.61)
USA major hurricane strikes	0.107 (*p* = 0.62)	−0.123 (*p* = 0.65)	−0.118 (*p* = 0.735)	−0.877 (*p* = 0.65)
Basin-wide Hurricane count recorded in HURDAT2	0.247 (*p* < 0.001)***	0.261 (*p* < 0.003)***	0.492 (*p* < 0.001)***	1.00 (*p* = 0.067)*
Basin-wide major hurricane count recorded in HURDAT2	0.718 (*p* < 0.001)***	0.601 (*p* < 0.001)***	0.710 (*p* < 0.001)***	1.96 (*p* = 0.026)**
Adjusted basin-wide hurricane count	−0.0855 (*p* = 0.16)	−0.0544 (*p* = 0.50)	0.194 (*p* = 0.070)*	N/A
Adjusted basin-wide major hurricane count	0.193 (*p* = 0.055)*	0.071 (*p* = 0.57)	0.105 (*p* = 0.52)	N/A

Trends in hurricane frequency measures over four different time periods, based on a Poisson regression to hurricane and major hurricane frequency with time as a covariate (see “Methods”); values are the time-dependent part of the rate parameter for a Poisson regression (see “Methods”). Counts are based on data from version 2 of the North Atlantic Hurricane Database (HURDAT2, ref. ^33^) and the adjustment developed in this study. Values with a *p-*value less than 0.1 are highlighted by a single asterisk (*), with a *p-*value less than 0.05 by a double asterisk (**), and less than 0.01 by a triple asterisk (***). We show four time periods because of key observing system inhomogeneities: 1851–1877 is the period before the U.S. Signal Corps was tasked with recording all tropical cyclones in the North Atlantic (ref. ^35^), 1900 is the time after which it is estimated that all hurricanes striking the United States of America (USA) would have been recorded (ref. ^36^), and homogenized satellite-based TC intensity data are available since 1980 (ref. ^14^).

In contrast to the frequency of HUs striking the USA, there is a clear and pronounced increase in the basin-wide NA HU and MH frequency recorded in the HURDAT2 database between 1851 and 2019 (Fig. 1c, d), with about triple the recorded NA MHs in recent decades compared to the mid-19th century. One possible interpretation of the distinct evolution of basin-wide and U.S.-striking HU and MH, is that U.S. strikes represent a fraction of the overall NA basin-wide frequency, and redistributions of HU activity within the NA basin could result in distinct evolutions of U.S. strikes and NA basin-wide frequency^37,38^. An additional or alternative contribution to the U.S. striking-to-basin-wide distinction could be that changing observing practices had a larger impact on basin-wide HU than on recorded U.S. HU strikes, leading to spurious increasing trends in recorded basin-wide HU^10^ and MH frequency. These possible explanations for the observed behavior are further explored below.

### Hurricane and major hurricane frequency adjusted for missing storms

Previous work has led to the development of a number of methods to estimate the impact of changing observing capabilities on the recorded increase in basin-wide HU frequency between 1878 and 2008 (ref. ^10^). We here update the analysis of ref. ^10^ to build an adjustment to recorded HU counts over 1851–1971, based on the characteristics of observed HUs over 1972–2019. We then extend that methodology to build an adjustment to recorded MH counts over 1851–1971, based on MHs recorded over 1972–2019 (see “Methods”). The methodology for the basin-wide count adjustment involves using HU (MH) tracks from an era we posit is fully sampled, along with ship-position data from the pre-fully-sampled era, to build a probabilistic estimate of the number of storms that may have occurred and not been detected in each year of the earlier era. There are a number of key assumptions that go into this methodology (see “Methods” section and refs. ^9,10^), including assuming that ships at sea and land would have been perfect observers, and that the types of TCs that have occurred in the fully sampled era are representative of those that could have occurred prior to the fully sampled period. After making these assumptions, and building a model for the radius of HU (≥33 m/s) or MH (≥ 50 m/s) winds, we construct our basin-wide NA HU and MH adjustment: the estimate of the time-evolving number of HUs or MHs that were likely missed before the early 1970s. Ref. ^9^ use 1966 as the start of the fully sampled era because at least once a day satellite pictures became routinely available: the sun-synchronous Environmental Science Services Administration (ESSA) satellites. However, the quality of these data is not sufficient to determine intensity (maximum wind) reliably, nor is there a systematic technique (Dvorak) calibrated for these data to obtain maximum winds. In 1972, high-resolution imagery from the Applications Technology Satellite (ATS) began to be used operationally, and the Dvorak technique was invented and used operationally during daylight hours on both the ESSA and ATS imagery, which were by then available electronically instead of fax-type imagery. However, we note that the main results in this study are not qualitatively altered by using 1966 as the start.

**Fig. 2:**
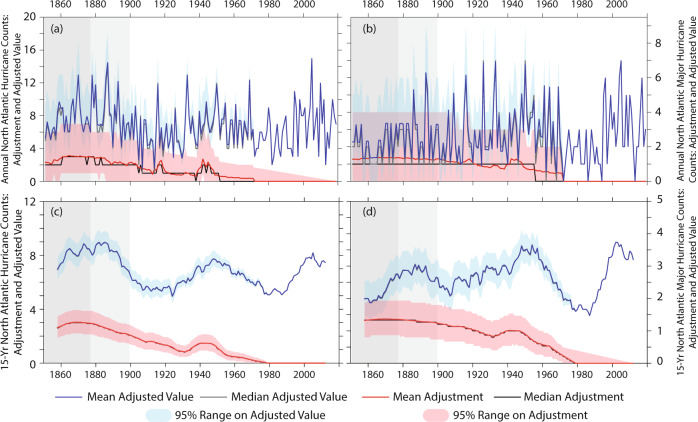
North Atlantic missing storm adjustment and adjusted basin-wide hurricane and major hurricane frequency. Top panels **a**, **b** show adjusted values for each year 1851–2019, bottom panels **c**, **d** show the values for the 15-year centered average of basin-wide frequency; the frequency of North Atlantic (NA) hurricanes is shown on the left and of NA major hurricanes on the right. In each panel, the red thick line shows the mean estimate of the missing storm adjustment, thick black line shows the median estimate, and the pink shading shows the 95% range on the adjustment based on a bootstrap resampling of the adjustments; thick blue line shows the mean estimate on basin-wide frequency (sum of frequency recorded in version 2 of the North Atlantic Hurricane Database, HURDAT2, ref. ^33^, and the adjustment developed in this study), thin black line shows the median estimate of the adjusted frequency, the sky-blue shading shows the 95% range on the adjusted value based on a bootstrap resampling of the adjustments. In the bottom panels **c**, **d** a dotted gray line shows the 15-year centered average of the recorded HURDAT2 counts (seen also in Fig. 1c, d). Gray background shading is as in Fig. 1, and highlights times where we have reduced confidence in the frequency estimates even after adjusting for likely missing storms.

The estimated number of missing NA HUs grows backward in time, reaching a peak value of ~3 HUs/year between 1860 and 1880 (red lines Fig. 2a, c); the updated reconstruction shows substantial similarity to that of ref. ^10^, which was based on satellite era HUs over 1966–2008. Meanwhile, the estimated number of missing NA MH shows a relatively steadier value for most of the record, at around one MH per year (red lines Fig. 2b, c). Additional robustness analysis leaving out sets of satellite era years^37^ shows that the HU and MH adjustments are not the result of particular satellite era years (Supplementary Material). For both MH and HU there is a local maximum in the annual correction centered around both World Wars—with the World War II maximum being evident even in the smoothed data (Fig. 2); these maxima in correction reflect a minimum in ship reports in the International Comprehensive Ocean Atmosphere Data Set  (ICOADS) during the World Wars. For frequency in a single year there is substantial uncertainty in both adjustments, so that it cannot be excluded at the 95% confidence level that no storms or that at a few times more than the central estimate were missed (pink shading in Fig. 2a, b). However, for the 15-year running smoothed counts the 95% range on the adjustment is smaller than for annual values, and the method indicates a significant undercount in both NA HUs and MHs for the entire pre-1960s period (pink shading in Fig. 2c, d)—we note that the results are qualitatively consistent for smoothing windows between 9- and 25-years.

Once the adjustment is added to the recorded number of Atlantic HUs and MHs, substantial year-to-year and decade-to-decade variability is still present in the data, with the late-19th, mid-20th and early-21st centuries showing relative maxima, and the early 20th and late 20th centuries showing local minima (Fig. 2). However, after adjustment, the recent epoch (1995–2019) does not stand out as unprecedented in either basin-wide HU or MH frequency. There have been notable years since 2000 in terms of basin-wide HU frequency, but we cannot exclude at the 95% level that the most active years in terms of NA basin-wide HU or MH frequency occurred in either the 19th century or mid-20th century (blue lines and shading in Fig. 2a, b). Further, we cannot exclude that the most active epoch for NA HU frequency was in the late-19th century, with the mid-20th century comparable to the early-21st in terms of basin-wide HU frequency. The 19th century maximum in activity is more pronounced in overall frequency than in MH frequency, while the late-20th century multi-decadal temporary dip in MH frequency stands out relative to that in the early-20th century. Relative to the satellite era and after adjustment, overall basin-wide frequency shows a more active late-19th century than does basin-wide MH frequency. Meanwhile, after adjustment the mid-20th century active period is more pronounced in basin-wide MHs than in overall HU frequency.

To evaluate secular changes in frequency, we build a Poisson regression for each of the HU and MH frequency records using time as a covariate (see Methods) and show the results in Table 1. We explore a number of start-dates for our trend estimate, and to assess the robustness of the trends, we also explore trends over 1980–2019 to place recent changes^14^ in the context of century-scale ones. The nominal century-scale decreases in the frequency of hurricanes striking the USA (both HU and MH) are generally not statistically significant, and differ from the 1980–2019 changes. However, the century-scale increases in HURDAT2 basin-wide HU and MH frequency are very significant and present for all start dates. However, once the missing storm adjustments are included, the nominal sign of the basin-wide HU trend changes for the early start dates, and is weakly significantly positive only for the 1900 start date. The adjusted basin-wide MH record retains a nominally positive trend, but the trends after 1878 are not significant, and those computed from 1851 are only marginally significant. Furthermore, the 1980–2019 increases in basin-wide HU and MH frequency are not a continuation of a longer-term trend, but reflect a recovery from a strong minimum in the 1970s and 1980s (Fig. 2)—this evolution suggests a dominant contribution to past multidecadal variations of HU and MH frequency from some combination of multi-decadal internal climate variability (such as Atlantic Multidecadal Variability tied to variations in the strength of meridional ocean heat transport in the Atlantic—refs. ^16–18^) and/or non-greenhouse gas forcing, such as variations in anthropogenic or natural aerosols^19–24^.

### USA hurricane strikes to basin-wide and MH/HU ratios

**Fig. 3:**
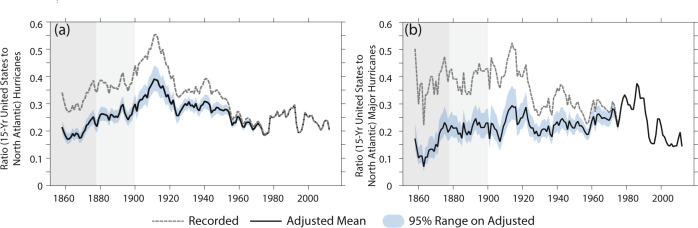
Ratio of USA hurricane strikes to basin-wide hurricane frequency. Ratio of the 15-year running count of United States of America (USA) strikes and 15-year running count of basin-wide frequency for hurricanes (**a**) and major hurricanes (**b**). Dotted gray line shows the values based on the recorded version 2 of the North Atlantic Hurricane Database (HURDAT2, ref. ^33^) frequency, while the thick solid line shows the value based on the HURDAT2 recorded USA strikes and the adjusted basin-wide frequencies; blue shading shows the 95% range on the ratio based on a Bootstrap sampling of the adjustment values. Gray background shading is as in Fig. 1, and highlights times where we have reduced confidence in the basin-wide and USA strike frequency estimates even after adjusting for likely missing storms.

In the raw HURDAT2 database, the century-scale evolution of recorded basin-wide NA HUs and MHs differs considerably from that of HUs and MHs striking the USA (compare top and bottom of Fig. 1). This difference results in a century-scale decrease in the fraction of basin-wide recorded overall and major hurricanes striking the USA (gray dotted lines Fig. 3), with about 40% of basin-wide MH striking the USA as a MH (Fig. 3b). One possible interpretation of this decreasing ratio is that there has been a century-scale shift in the tracks of HU and MH, or that in recent decades HUs and MHs are losing either their intensity or tropical nature as they approach the coast of the USA^38,39^. An alternative interpretation is that USA HU and MH strikes have been better observed since the mid-1850s than basin-wide frequency of either, resulting in a spurious inflation of the USA strike-to-basin-wide ratio in the pre-satellite era^40^. The adjusted basin-wide HU and MH records support the latter hypothesis: once we include the adjustment for likely missing storms, there is no longer a clear century-scale decrease in this ratio (black line in Fig. 3).

We can assess secular changes in the fraction of basin-wide HUs and MHs that strike the USA using a Binomial regression model with time as a covariate (top four rows of Table 2, see “Methods”). For the HURDAT2 data, the century-scale decreases in USA-striking proportion are very significant (top row Table 2). After adjusting for missing storms, the century-scale decrease in USA-striking HU fraction is weaker and of modest significance, largely reflecting the influence of a maximum in the 1910s (Fig. 3). However, the century-scale changes in USA-striking MH fraction do not show any significant secular change, with around 20–30% of NA MHs over 15-year periods having struck the USA as MHs. Based on our adjusted estimates, it appears that the stationary ratio of USA-striking to basin-wide MHs reported over the late-20th century (ref. ^41^) is evident since the mid-19th century, and we do not see evidence for strong multi-decadal modulation of the USA-striking MH fraction^40^.

**Table 2:** Measures of secular change in USA-strike to basin-wide frequency, and in ratio of MH to HU frequency.

	Time-dependence of the probability parameter (µ) in the Binomial regression (1/century)			
	1851–2019	1878–2019	1900–2019	1980–2019
USA hurricane Strikes vs. HURDAT2 basin-wide hurricanes	−0.541 (*p* < 0.001)***	−0.774 (*p* < 0.001)***	−1.02 (*p* < 0.001) ***	−0.602 (*p* = 0.65)
USA major hurricane strikes vs. HURDAT2 basin-wide major hurricanes	−0.934 (*p* *<* 0.001)***	−1.01 (*p* = 0.0021)***	−1.95 (*p* = 0.006) ***	−4.151 (*p* = 0.081)*
USA hurricane strikes vs. Adj. basin-wide hurricanes	−0.0677 (p = 0.62)	−0.316 (*p* = 0.071)*	−0.533 (*p* = 0.028) **	N/A
USA major hurricane strikes vs. Adj. basin-wide major hurricanes	−0.116 (*p* = 0.64*)*	−0.246 (*p* = 0.42)	−0.282 (*p* = 0.47)	N/A
USA major hurricane strikes vs. USA hurricane strikes	0.376 (*p* = 0.16)	0.266 (*p* = 0.41)	0.122 (*p* = 0.78)	−2.43 (*p* = 0.33)
HURDAT2 basin-wide major hurricanes vs. basin-wide hurricanes	0.677 (*p* < 0.001)***	0.497 (*p* = 0.0052)***	0.360 (*p* = 0.13)	1.70 (*p* = 0.14)
Adj. basin-wide major hurricanes vs. basin-wide hurricanes	0.424 (p < 0.001)***	0.193 (*p* = 0.22)	−0.153 (*p* = 0.47)	N/A

Values are the time-dependent parameter for the probability estimated from a Binomial regression (see “Methods”). First four rows show trends in the fraction of basin-wide frequency striking the United States of America (USA) over four different time periods, last three rows show trends in the fraction of hurricane (HU) frequency that are major hurricanes (MH) over four different time periods, based on a Binomial regression for MH frequency as a subset of HU frequency with time as a covariate (see “Methods”). Counts are based on data from version 2 of the North Atlantic Hurricane Database (HURDAT2, ref. ^33^) and the adjustment developed in this study. Values with a *p-*value less than 0.1 are highlighted by a single asterisk (*), with a *p-*value less than 0.05 by a double asterisk (**), and less than 0.01 by a triple asterisk (***). Time periods shown as in Table 1.

**Fig. 4:**
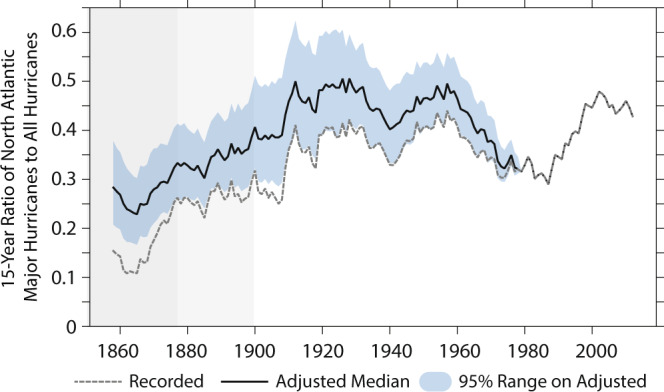
Ratio of North Atlantic major hurricanes to overall hurricanes. Ratio of the 15-year running count in North Atlantic basin-wide major hurricane to hurricane frequency. Gray line shows data based on version 2 of the North Atlantic Hurricane Database (HURDAT2, ref. ^33^) recorded frequencies, while the blue line shows the ratio based on the mean adjusted frequencies (light blue shading shows the 95% range based on a Bootstrap resampling of the adjustment). Gray background shading is as in Fig. 1, and highlights times where we have reduced confidence in the frequency estimates even after adjusting for likely missing storms.

In estimates of the sensitivity of NA HU activity to greenhouse-induced warming and 21st century projections based on dynamical or statistical-dynamical models^6,20–22,25–31^, there is more consistency for an increase in the *fraction* of HUs becoming MHs (that is, an intensification of HU) than in either the overall frequency of HUs or MHs. In the raw HURDAT2 dataset, there is a substantial century-scale increase in the NA MH/HU ratio since the late-1800s (gray line in Fig. 4). However, once the adjustment is added to both NA HUs and MHs (blue line and shading in Fig. 4), the running 15-year MH/HU ratio is dominated by multi-decadal fluctuations, with minima of 25–30% in the mid-1850 s and in the decades centered around the 1980s, and maxima of 40–50% in the early-to-mid-20th century and the early 21st century. The low values in the 1850–1878 period, while being unique in the record, also occur during the period when we have least confidence in the data—based on these considerations, we view with skepticism any century-scale trend that arises only once the 1850–1878 period is included. In our adjustment methodology, we assume that ships at sea do not aim to steer away from HU and MH winds (Assumption 5, “Methods” section)—this assumption may be less justified for MH winds, and may result in an underestimate of MH relative to HU in the record even after adjustment. Nevertheless, the recent increase in the proportion of NA HUs becoming MHs, after adjustment, which is also reflected in the results of ref. ^14^, is not a continuation or acceleration of a long-term trend, but rather is a rebound from a deep minimum in the decades surrounding the 1980s—see below for a discussion of possible mechanisms.

We evaluate secular changes in the fraction of HUs becoming MHs through a Binomial regression model with time as a covariate (bottom three rows of Table 2; see “Methods”). The fraction of HUs striking the USA as MHs does not show a significant change for any of the epochs we explore. For both HURDAT2 and the adjusted series, there is a significant increase in basin-wide MH fraction over 1851–2019. The HURDAT2 series shows at least a nominal increase in MH fraction for all the epochs explored, though the *p*-value exceeds 0.1 for the 1900–2019 and 1980–2019 periods. Meanwhile, for the adjusted MH and HU records, the trends in basin-wide MH fraction are neither significant nor of consistent sign for 1878–2019 and 1900–2019. After adjustment of the basin-wide MH and HU record, century-scale increases in basin-wide MH frequency depend on the pre-1878 era, before the U.S. Signal Corps started efforts to monitor all Atlantic HUs^35^.

## Discussion

One of the most consistent expectations from projected future global warming is that there should be an increase in TC intensity, such that the fraction of MH to HU increases^6,20–22,25–31^. This issue has become more pressing with the recent finding of a global increase in this metric since 1979 using homogenized satellite-based data^14^—a finding to which Atlantic HU contribute. We here build on the methods of refs. ^9,10^ to build a homogenized record of Atlantic MH frequency and MH/HU ratio since the 19th century. We find here that, once we include a correction for undercounts in the pre-satellite era basin-wide NA HU and MH frequency, there are no significant increases in either basin-wide HU or MH frequency, or in the MH/HU ratio for the Atlantic basin between 1878 and 2019 (when the U.S. Signal Corps started tracking NA HUs^35^). We suggest that the modestly significant 1851–2019 increase in basin-wide MH frequency and MH/HU ratio that remains after including the HU and MH adjustment reflects data inhomogeneity that our adjustment is unable to correct—rather than an actual increase in these quantities. The homogenized basin-wide HU and MH record does not show strong evidence of a century-scale increase in either MH frequency or MH/HU ratio associated with the century-scale, greenhouse-gas-induced warming of the planet. For example, the temporal evolution of the global mean temperature is not closely reflected in the temporal evolution of adjusted MH/HU ratio shown in Fig. 4.

Does this work provide evidence *against* the hypothesis that greenhouse-gas-induced warming may lead to an intensification of North Atlantic HUs? Not necessarily. Substantial multi-decadal variability may obscure trends computed over the past century^16–18,20,21^, and recent studies suggest the possibility for an aerosol-driven reduction in NA HU and MH activity over the 1960s–1980s (refs. ^19–24^), which may have obscured any greenhouse induced NA HU and MH intensification over the 20th century. For example, a statistical downscaling of global climate models (GCMs) that were part of the Coupled Model Intercomparison Project Phase 5 (CMIP5) shows a robust and significant projection for a greenhouse gas-induced 21st century NA hurricane intensification; yet applying that same method to historical simulations the greenhouse-induced intensification over the late-19th and 20th century is masked by the late-20th century aerosol-induced weakening^20^. Historical simulations show that aerosol forcing may have masked the 19th-20th century greenhouse-gas-induced increase in potential intensity, the theoretical upper bound on tropical cyclone intensity, even though climate models show increases in potential intensity in tropical cyclone regions in response to projected future warming^24–26^. The homogenized MH and HU data developed in the present study serve as a target for century-scale historical simulations with high-resolution dynamical and statistical models that are used for 21st century projections.

The adjusted NA basin-wide MH frequency and MH/HU ratio show substantial multi-decadal variability (Figs. 2, 4), and the adjusted basin-wide MH frequency shows its lowest values over the 1960s–1980s (Fig. 2). These features show at least qualitative consistency with the notion of a strong influence of either internal multi-decadal climate variability and/or late-20th century aerosol-induced weakening of NA HU intensity during that period. Our homogenized records also correspond with document- and proxy-based reconstructions of Antilles and Atlantic HUs, which indicate that substantial variability in HU frequency has been present in the Atlantic, and the inactive period in the late 20th century may have been the most inactive period in recent centuries^42,43^.

The homogenized hurricane records suggest a consistent and marginally statistically significant decrease in the ratio of basin-wide hurricanes striking the USA as hurricanes (Table 2, row 3). Some models project an eastward shift in the location of NA TCs in response to increasing greenhouse gases (e.g., refs. ^27,28^), so this observed change may reflect the emerging impact of greenhouse warming on NA TC tracks. However, although there is a nominal decrease in the ratio of basin-wide MH striking the USA as MH (Table 2, row 4), the trends are not significant for any of the time periods explored.

Caution should be taken in connecting recent changes in Atlantic hurricane activity to the century-scale warming of our planet. The adjusted records presented here provide a century-scale context with which to interpret recent studies indicating a significant recent increase in NA MH/HU ratio over 1980–2017 (ref. ^14^), or in the fraction of NA tropical storms that rapidly intensified over 1982–2009 (ref. ^15^). Our results indicate that the recent increase in NA basin-wide MH/HU ratio or MH frequency is not part of a century-scale increase. Rather it is a rebound from a deep local minimum in the 1960s–1980s. We hypothesize that these recent increases contain a substantial, even dominant, contribution from internal climate variability^16–18,20,21^, and/or late-20th century aerosol increases and subsequent decreases^19–24^, in addition to any contributions from recent greenhouse gas-induced warming^20,22,24,44^. It has been hypothesized, for example, that aerosol-induced reductions in surface insolation over the tropical Atlantic since between the mid-20th century and the 1980s may have resulted in an inhibition of tropical cyclone activity^19–24^; the relative contributions of anthropogenic sulfate aerosols, dust, and volcanic aerosols to this signal (each of which would carry distinct implications for future hurricane evolution)—along with the magnitude and impact of aerosol-mediated cloud changes—remain a vigorous topic of scientific inquiry. It has also been suggested that multi-decadal climate variations connected to changes in meridional ocean overturning may have resulted in a minimum in northward heat transport in the Atlantic and a resulting reduction in Atlantic hurricane activity^16–18,20,21^. Given the uncertainties that presently exist in understanding multi-decadal climate variability, the climate response to aerosols and impact of greenhouse gas warming on NA TC activity, care must be exercised in not over-interpreting the implications of, and causes behind, these recent NA MH increases. Disentangling the relative impact of multiple climate drivers on NA MH activity is crucial to building a more confident assessment of the likely course of future HU activity in a world where the effects of greenhouse gas changes are expected to become increasingly important.

## Methods

### Missing storm adjustment methodology

We extend the methodology described in refs. ^9,10^ to NA overall HU frequency since 1851, and adapt the methodology to NA major (Saffir-Simpson Category 3–5) hurricane frequency since 1851. For North Atlantic HU frequency, the methodology is that of ref. ^10^, except we use a longer HURDAT2 dataset^34^: from 1972 to 2019, instead of the 1972–2008 record used in ref. ^10^ to develop the correction. We also extend the recount estimates to span the full HURDAT2 record of 1851–1971, instead of 1878–1971 as was done in Refs. ^9–12^.

Using the methodology for HU adjustment of ref. ^10^, the undercount adjustment is developed using an observing system emulation, in which we compare HU tracks from the satellite era (1972-present) to ship track density from the International Comprehensive Ocean-Atmosphere Data Set (ICOADS, ref. ^45^) dataset from the pre-satellite era (1851–1971). The probability that a given storm from the satellite era would have been missed had it occurred in a particular pre-satellite year is estimated through an ensemble by sampling across 21 different shifts in the storm’s actual date of occurrence (shifting forward and backward in the calendar by 0, 5, 10, 15, …, 45, 50 days), and by drawing 100 realizations of the radius of gale-force and hurricane strength winds from a probability density function (PDF) based on the observations of ref. ^46^. For each realization, we assess that a HU would have been detected if either one land observation would have been within the parameterized radius of hurricane winds (R33), or two ship observations would have been within the model-parameterized radius of tropical storm winds (R17), with at least one being withing radius of hurricane-force winds (R33). We also require that the first detection of a tropical storm or HU must be equatorward of 40°N. Radius of 17 and 33 ms^−1^ winds (R17 and R33) are parameterized based on the data of ref. ^46^, the radii are multiplied by 0.85 to correct from maximum extent to mean extent.

The average radius of tropical storm winds (R17) is parameterized such that the logarithm of the radius follows a normal distribution, with the random seed selected for each storm. As reported in ref. ^9^, R17 (in kilometers) is parameterized based on the wind speed of the storm (*v*_max_) as follows, where *ξ* is a normally distributed random variable for each storm with a mean of zero and a standard deviation of one:

1 $${\rm{R}}17=0.85\ast \left\{\begin{array}{cc}0 & {v}_{\max } < 17\,{\rm{ms}}^{-1} \\ 90{e}^{\xi /1.3}+70 & \;\;\;\;\;\;\;\;\;\,\;\;\;\;\;17\,{\rm{ms}}^{-1} \le v_{\max } < 33\,{\rm{ms}}^{-1} \\ 90{e}^{\xi /1.3}+150 & \;\;\;\;\;\;\;\;\;\,\;\;\;\;\;33\,{\rm{ms}}^{-1}\le v_{\max } < 50\,{\rm{ms}}^{-1}\\ 90{e}^{\xi /1.3}+170 & 50\,{\rm{ms}}^{-1}\le v_{\max }\end{array}\right.$$

The average radius of hurricane winds (33 ms^−1^) winds is parameterized such that the logarithm of the radius follows a normal distribution when the storm winds exceed 33 ms^−1^, and is zero when the storm is weaker than hurricane strength, using the parameterization of ref. ^10^, where *ξ* is a normally distributed random variable for each storm with a mean of zero and a standard deviation of one:

2 $${\rm{R}}33=0.85\ast \left\{\begin{array}{cc}0 & {v}_{\max} < 33\,{\rm{ms}}^{-1}\\ 90{e}^{\xi /2.1}-15 & \;\;\;\;\;\;\;\;\;\,\;\;\;\;\;33\,{\rm{ms}}^{-1}\le v_{\max} < 50\,{\rm{ms}}^{-1}\\ 90{e}^{\xi /2.1}+5 & 50\,{\rm{ms}}^{-1}\le v_{\max }\end{array}\right.$$

The probability of a satellite era storm being detected is computed as the total realizations in which the storm was detectable divided by the total realizations in a given pre-satellite observing system year (21 date shifts × 100 size ensembles = 2100). The mean missing storm count estimate for a given pre-satellite era year is the sum across all satellite era years of the sums of the probability the storms were missed (that is 1 minus the probability that it would have been detectable in a given year had it occurred). We build a Bootstrap uncertainty estimate for the missing storm counts by drawing 10,000 samples (with replacement) for each pre-satellite era year from the 2100 ensembles × 48 satellite era years = 100,800.

For MHs, the methodology of ref. ^10^ is adapted by changing the detection threshold to be a single ship or a single land point within the modeled radius of 50 ms^−1^ winds (see below). We do not require multiple 50 ms^−1^ detections, nor do we place a latitude threshold on the detection. Furthermore, we assess that the pre-satellite era for MHs is likely 1851–1971, rather than 1851–1965 - although only computing the correction over the period 1851–1965 does not affect any of the principal results of this study. The probability of a satellite era MH being detected is computed in an analogous manner to that for overall HU frequency, generating an ensemble by shifting the timing of satellite era storms and producing multiple realizations of 50 ms^−1^ radius.

### Major hurricane wind radius model

To build a model for the radius of 50 ms^−1^ winds (R50), we use the HWIND 1998–2013 estimates of wind radii^47^. We build the model using the observations that meet the requirements during the period (1998–2013). Note that one MH can have multiple MH observations during its lifespan. For each observation, we identify the location(s) where the wind speed exceeds 50 ms^−1^ and calculate the distance from the HU center to the location(s). The radius of 50 ms^−1^ winds for a HU observation is the averaged distance from the HU center to the locations with exceeding 50 m s^−1^ wind speed. We fit the lognormal distribution (i.e., µ = 3.416, σ = 0.478) for the radii of 243 MH observations during the study period. Therefore, the R50 parameterization is as follows, where *ξ* is a Normally distributed random variable for each storm with a mean of zero and a standard deviation of one:

3 $${\rm{R}}50=\left\{ \begin{array}{cc}0 & {v}_{\max } < 50\,{\rm{ms}}^{-1}\\ {e}^{3.416+0.478\xi } & 50\,{\rm{ms}}^{-1}\le v_{\max }\end{array}\right.$$

### Key assumptions in the hurricane adjustment methodology

The key assumptions in the HU adjustment methodology are discussed at greater length in Refs. ^9,10^, but we briefly list them here for the benefit of the reader:

1. All land points and ship observations are perfect storm detectors: this will bias the storm adjustment low, particularly in the 1800s.
2. Ship tracks in the ICOADS database^44^ are representative of ships that have provided meteorological data for storm identification^34^ this will bias the storm adjustment high if there is considerable other independent data available. We note that we include all ICOADS observations, regardless of the meteorological data reported, which could overestimate the data available for storm identification, which should partially mitigate the bias.
3. All storms detectable by the ships have been, or will be, included in HURDAT2: this will bias the storm adjustment low.
4. TCs are assumed radially symmetric: this will likely lead to random adjustment errors, rather than a systematic bias.
5. Ships and land can perfectly measure storm wind (at least to the threshold for HU or MH identification): if there is a systematic under- (over-)estimation of winds, this will lead to an under- (over-)estimation of historical frequency.
6. Ships did not attempt to, or were unable to, avoid storms: this assumption leads to an underestimate of the adjustment.
7. Modern era storm tracks are representative of the storm tracks that could have occurred in the pre-satellite era: errors in this assumption will lead to reductions in any real variations and changes in HU and MH activity. This would also lead to underestimates in the time-smoothed uncertainty estimates.
8. Sufficient information in addition to wind speed would be available to identify a HU or MH, if HU or MH winds are observed: this leads to an underestimate in the adjustment.
9. Assume that single HU or MH events were not inaccurately counted as multiple systems in HURDAT2: if this happened the storm count for that period would be biased high, all other factors equal

### Trend measures

To measure the secular trend in the various measures of aggregate NA HU and MH activity, we fit statistical models using time as a covariate. For frequency statistics (e.g., the number of HUs or MHs striking the USA, and HU and MH basin-wide frequency), we model the counts through a Poisson regression model, such that the probability distribution of the annual count (*N*_*x*_) for each frequency metric (*x*; e.g., USA HU strikes, basin-wide MHs) is:

4 $$p(N_{x}=k|\lambda)= \frac{\lambda_{x}(t)^{k}e^{-{\lambda}_{x}(t)}}{k!}{\rm{for}}\,k=0,1,2$$

for which we use the available data for each quantity and assume that the rate of occurrence (*λ*_*x*_*(t)*) is a function of time through a logarithmic link function:

5 $$\lambda_{x}(t)={e}^{{a}_{x}+b_{x}t}$$

where *t* is time (measured in (years C.E/100), *a*_*x*_ gives a measure of the base rate and *b*_*x*_ gives a measure of the time dependence of the rate or the trend measure, for each frequency measure *x* (e.g., USA HU strikes, basin-wide MH). To summarize the time dependence of the rate parameter (trend), we show in Table 1 the time-dependent coefficient (*b*_*x*_*)* of the rate parameter of the Poisson regression (*λ*_*x*_*(t)*).

For ratio statistics (e.g., the MH/HU ratio), we model the counts through a Binomial distribution, such that the probability distribution of the annual count of the subset variable (*N*_*y*_) for each frequency ratio metric (*N*_*y*_*/N*_*x*_; e.g., MH/HU ratio) is:

6 $$p({N}_{y}=k{\rm{|}}{\mu }_{x,y}(t),{N}_{x})=\frac{\varGamma ({N}_{x}+1){\rm{\cdot }}\varGamma ({N}_{y}+1)}{\varGamma ({N}_{x}-{N}_{y}+1)}{\mu }_{x,y}^{k}{(1-{\mu }_{x,y})}^{{N}_{x}-k}\;{\rm{for}}\,{k}=0,1,2\ldots {\it{N}}{\it{x}}$$

for which we use the available data for each quantity to fit the probability of success (*µ*_*x,y*_*(t)*) as a function of time, through a logistic link function:

7 $${\mu}_{x,y}(t)=\frac{1}{1+{e}^{-(a_{x,y}+b_{x,y}t)}}$$

where *t* is time (measured in (years C.E. /100), *a*_*x,y*_ gives a measure of the base probability and *b*_*x,y*_ gives a measure of the time dependence of the probability, or the trend measure, for the ratio of each frequency measure (*N*_*y*_*/N*_*x*_, e.g., MH/HU ratio). To summarize the time dependence of the probability (trend), we show in Table 2 the time-dependent coefficient (*b*_*x,y*_) of the probability of the Binomial regression (*μ*_*x,y*_*(t)*).

The Poisson and Binomial regression fit calculations are performed in *R* (ref. ^48^) using the freely available gamlss package (ref. ^49,50^). In Tables 1 and 2, we report the values of the trend factor in the regressions (*b*_*x*_ for the Poisson regression and *b*_*x,y*_ for the Binomial regression), along with the *p-*value of the time-dependent coefficient (*b*_*x*_ or *b*_*x,y*_) estimated using the gamlss package.

## Supplementary information

Supplementary Materials

## Data Availability

The median hurricane and major hurricane adjustments from 1851 to 2019 developed in this study, along with the 10,000-member Bootstrap resampling of each, are made freely and publicly available at the Department of Geosciences collection of the Princeton University DataSpace: 10.34770/epch-0h54.

## Code availability

Scripts and data used to generate figures are made freely and publicly available at the Department of Geosciences collection of the Princeton University DataSpace: 10.34770/epch-0h54.
