# Supplementary material for: Changes in Atlantic major hurricane frequency since the late-19th century
Source: Nat Commun. 2021 Jul 13;12:4054. doi: 10.1038/s41467-021-24268-5 (PMC8277888; doi:10.1038/s41467-021-24268-5)
Supplement: Supplementary file 1 — Supplementary Materials [file 41467_2021_24268_MOESM1_ESM.docx]

**Supplementary Material to: Changes in Atlantic Major Hurricane Frequency Since the Late-19th Century**

Submitted to *Nature Communications*

*Revision 1*

Gabriel A. Vecchi^1,2^, Christopher Landsea^3^, Wei Zhang^4^, Gabriele Villarini^5^, Thomas Knutson^6^

1. Geosciences Department, Princeton University, Princeton, NJ, USA
2. High Meadows Environmental Institute, Princeton University, Princeton, NJ, USA
3. National Hurricane Center, National Weather Service, National Oceanic and Atmospheric Administration, Miami, FL, USA
4. Department of Plants, Soils and Climate, Utah State University, Logan, UT, USA
5. IIHR-Hydroscience & Engineering, University of Iowa, Iowa City, IA, USA
6. Geophysical Fluid Dynamics Laboratory, National Oceanic and Atmospheric Administration, Princeton, NJ, USA

Corresponding Author: Gabriel A. Vecchi, Princeton University, Princeton, NJ, 08540, USA. [gvecchi@princeton.edu](mailto:gvecchi@princeton.edu), 609-258-7813

In order to evaluate the robustness of the estimate for Atlantic hurricane (HU) and major hurricane (MH) undercounts reported in the main text to satellite era years used in training, we have performed a sensitivity analyses inspired by K-fold cross validation (Elsner and Jagger 2013). Since we have 48 satellite era years on which we train the adjustment (1972-2019), it is natural to explore *K=6, 8 and* 12 robustness analysis. For each *K* (6, 8 and 12), we leave out *K* contiguous sets of *48/K* satellite-era years (*e.g.*, for *K=*8, we use 1972-1977 through 2014-2019) from our calculation of the missing storm adjustment. This allows us to evaluate the sensitivity of the mean adjustment to the satellite era years used. The results for *K=*8, 6 and 12 are shown, respectively, in Supplementary Figures 1-3 below. Although there are slight variations in the mean estimate of the HU and MH adjustment evident in the various *K-*fold robustness estimates, all of the mean estimates are qualitatively consistent and lie well within the Bootstrap estimated uncertainties, and none of the principal results discussed in the manuscript is altered. These estimates indicate an expected ~3 HU/year and ~1.2 MH/year unobserved in the late 19^th^ century.


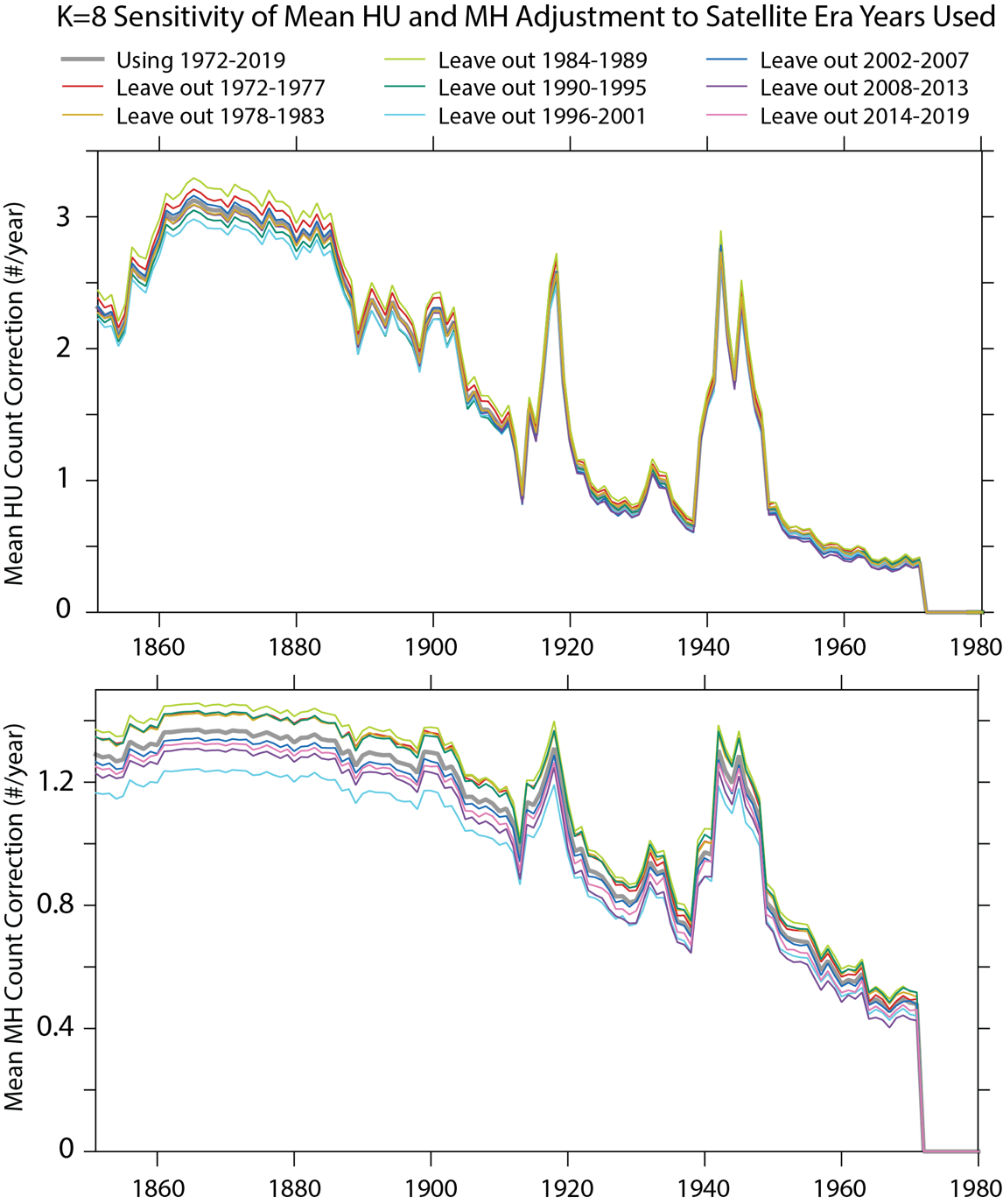


**Supplementary Figure 1: K=8-fold robustness analysis of the HU and MH adjustments.** Analysis of the sensitivity of the mean hurricane (upper panel) and major hurricane adjustments (lower panel) to leaving out eight different sets of satellite-era years in their development. The thick gray line shows the mean missing storm adjustment using all satellite-era years 1972-2019. Each colored line is the result of a K=8-fold sensitivity analysis (Elsner and Jagger 2013), in which K=8 contiguous segments of six years each are removed from the satellite era data before computing the count adjustments..


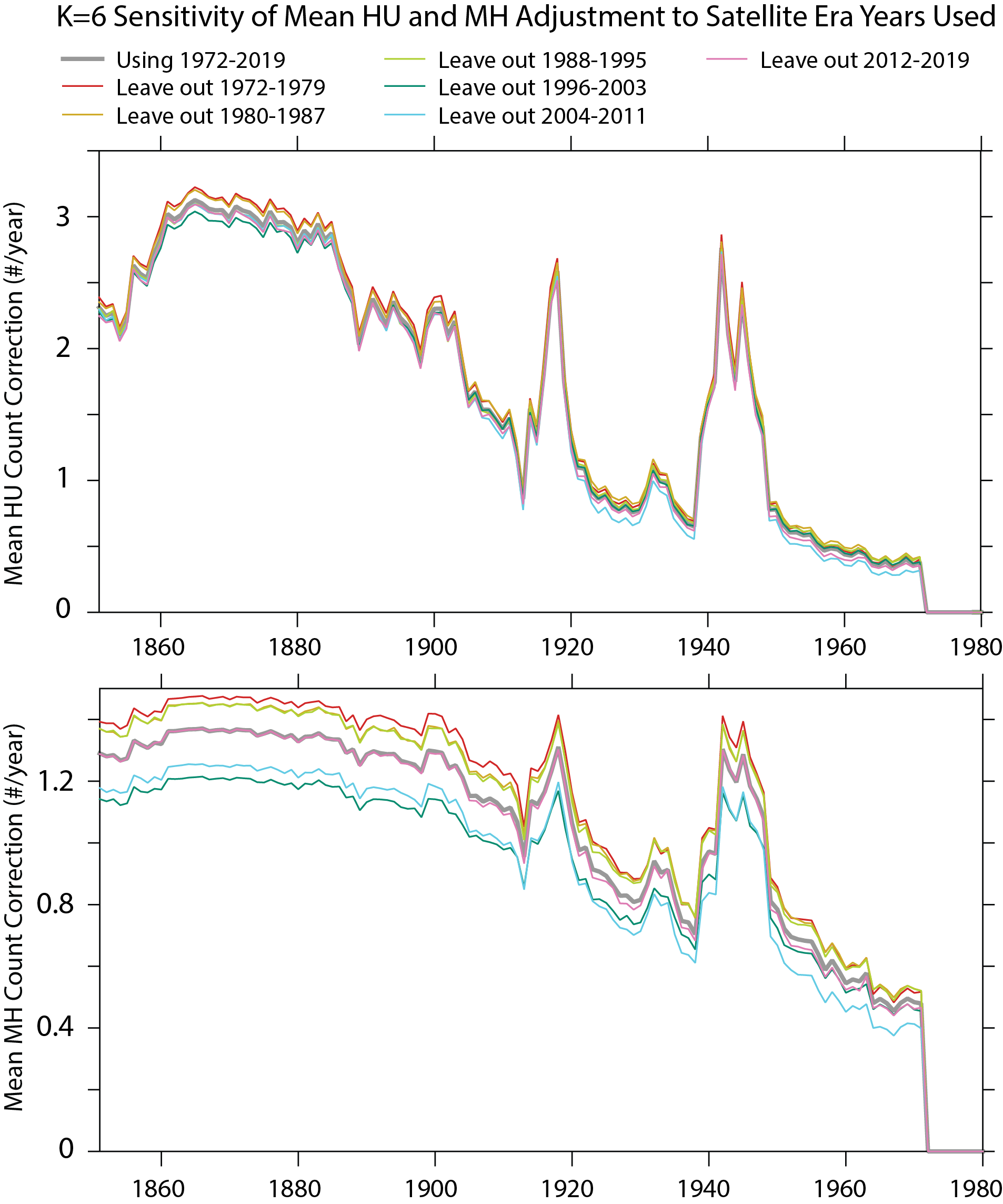


**Supplementary Figure 2: K=6-fold robustness analysis of the HU and MH adjustments.** Analysis of the sensitivity of the mean hurricane (upper panel) and major hurricane adjustments (lower panel) to leaving out six different sets of satellite-era years in their development. The thick gray line shows the mean missing storm adjustment using all satellite-era years 1972-2019. Each colored line is the result of a K=6-fold sensitivity analysis (Elsner and Jagger 2013), in which six contiguous segments of eight years each are removed from the satellite era data before computing the count adjustments..


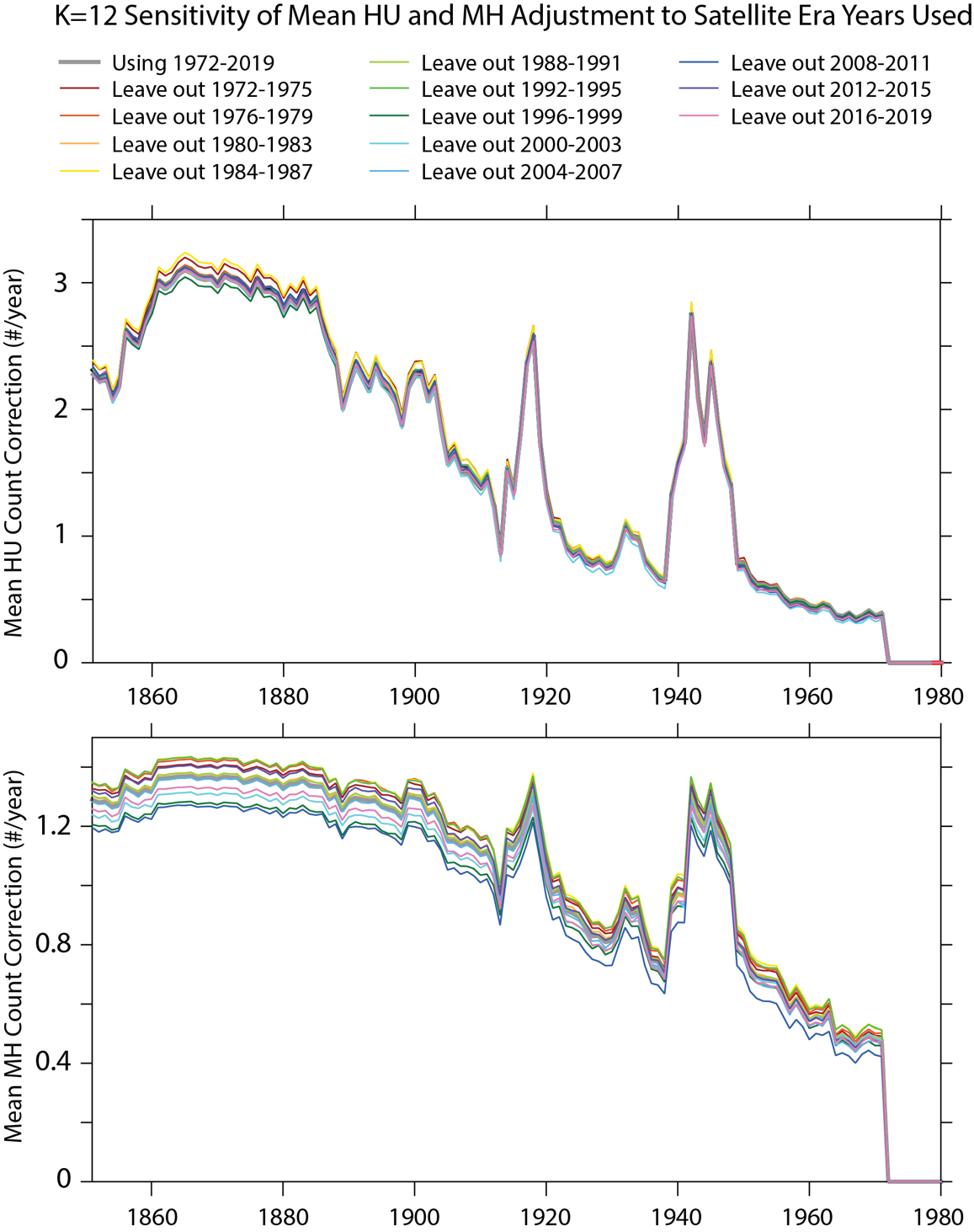


**Supplementary Figure 3: K=12-fold robustness analysis of the HU and MH adjustments.** Analysis of the sensitivity of the mean hurricane (upper panel) and major hurricane adjustments (lower panel) to leaving out twelve different sets of satellite-era years in their development. The thick gray line shows the mean missing storm adjustment using all satellite-era years 1972-2019. Each colored line is the result of a K=12-fold sensitivity analysis (Elsner and Jagger 2013), in which twelve contiguous segments of four years each are removed from the satellite era data before computing the count adjustments.

**References**:

1. Elsner, J. B. and T. H. Jagger, 2013: Frequency models. Hurricane Climatology: A Modern Statistical Guide Using R, Oxford University Press.
